# Targeting Oxidative Stress in Diabetic Complications: New Insights

**DOI:** 10.1155/2018/1909675

**Published:** 2018-06-19

**Authors:** Hao Wu, Lu Cai, Judy B. de Haan, Robertina Giacconi

**Affiliations:** ^1^Department of Nephrology, The Second Hospital of Jilin University, Changchun, Jilin, China; ^2^Pediatric Research Institute, Departments of Pediatrics, Radiation Oncology, and Pharmacology and Toxicology, University of Louisville, Louisville, KY, USA; ^3^Baker Heart and Diabetes Institute, Melbourne, VIC, Australia; ^4^Advanced Technology Center for Aging Research, Scientific and Technological Pole, Italian National Institute on Health and Science of Aging (INRCA), Ancona, Italy

Diabetes is a serious disease, and the number of adults affected by diabetes is predicted to grow worldwide from 422 million (estimated number in 2014) to 642 million by 2040. Diabetes causes high morbidity and mortality through its complications, with oxidative stress known to be a major contributor. The present special issue, which includes 3 original research articles and 4 review papers, is devoted to novel insights into targeting oxidative stress in diabetic complications.

New strategies designed to attenuate diabetes-induced oxidative stress, through the blockage of reactive oxygen species (ROS) generation and the enhancement of antioxidant-scavenging activity, are revealed in the original research articles published in this special edition. NADPH oxidase 4 (NOX4) is a key factor that generates ROS and contributes to the pathogenesis of diabetic kidney disease (DKD). Inhibition of NOX4 improves DKD. In the report by F. Hu et al., early growth response protein 1 (EGR1) was found to be a transcription factor involved in the regulation of the *Nox4* gene and responsible for the diabetes-induced renal oxidative stress. This study highlights that EGR1 is a potential novel target in future management of DKD. Diabetes-induced advanced glycation end products (AGE) lead to aberrant angiogenesis in the brain. The study by A. Alhusban et al. showed that silymarin, a milk thistle seed extract, attenuated AGE-induced aberrant angiogenesis by glycogen synthase kinase-3*β*-mediated inhibition of VEGF release. In addition to blocking ROS generation, activation of the nuclear factor-erythroid-2-related factor 2 (NRF2) antioxidant pathway is another effective approach. K. Shukla et al. reported that the aldose reductase inhibitor fidarestat alleviated oxidative stress during hyperglycemic stress via activation of NRF2 signaling, the effect of which enhanced the antioxidant-scavenging capacity.

The review articles in the present special issue have updated the findings of several complications of diabetes, such as cardiomyopathy, nephropathy, polyneuropathy, and cognitive dysfunction. B. Yan et al. have provided an overview of antioxidant natural products in the prevention of diabetic cardiomyopathy, with an emphasis being on key targets of natural products. Y. Yang et al. reviewed the effect of serotonin and its receptor on the induction of oxidative stress in DKD, suggesting the targeting of this axis as a therapeutic strategy for DKD and macrovascular complications. The review by S. Sifuentes-Franco et al. summarized the role of oxidative stress in diabetic polyneuropathy. Finally, Y. Cao et al. reviewed the multiple functions of sirtuin 1 in diabetic cognitive dysfunction, including its antioxidant activity.

Collectively, the present special issue reviews previous work and provides new findings in diabetic complications, with an emphasis on targeting oxidative stress. We hope that this special issue brings new insight for future management of diabetic complications.

